# The C-terminus of SPE-11 is required for proper embryonic development in *C. elegans*

**DOI:** 10.17912/micropub.biology.000255

**Published:** 2020-05-21

**Authors:** Aimee Jaramillo-Lambert, Andy Golden

**Affiliations:** 1 National Institute of Diabetes and Digestive and Kidney Diseases, National Institutes of Health, Bethesda, Maryland 20892; 2 Presently-Department of Biological Sciences, University of Delaware, Newark, Delaware 19701

**Figure 1:**
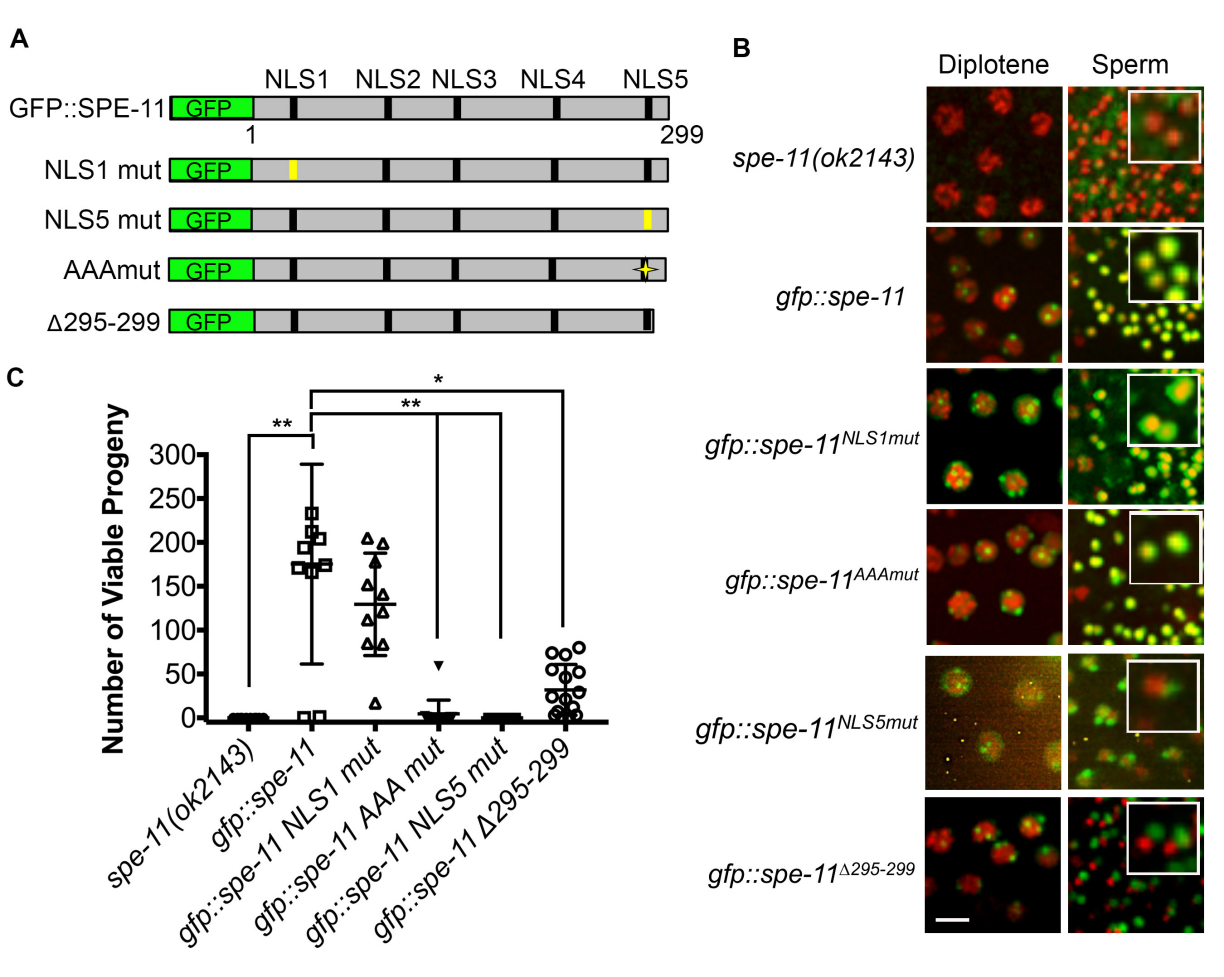
**C-terminal mutations of SPE-11 fail to rescue a *spe-11* deletion mutant**. **A**. Cartoon of SPE-11 constructs for *in vivo* localization studies and functional analyses. All constructs were expressed as GFP fusions from the *spe-11* promoter and 3’UTR. Putative nuclear localization signals (NLS) are indicated by vertical black bars. Mutated NLSs are indicated by vertical yellow bars. A yellow star for the GFP::SPE-11^AAA^ mutant indicates the following amino acid changes: K289A, R292A, and K293A. **B**. Transgenic males expressing different GFP::SPE-11 (green) fusion constructs and mCherry-histone (red) were analyzed for proper localization in the *spe-11(ok2143*) mutant in diplotene nuclei and post-meiotic sperm. Insets are enlargements of post-meiotic sperm. Scale bar, 5 µm. **C**. The number of viable progeny produced by *spe-11(ok2143*) and *spe-11(ok2143*) expressing *gfp::spe-11*, *gfp::spe-11^NLS1mut^*, *gfp::spe-11^AAAmut^, gfp::spe-11^NLS5mut^,* or *gfp::spe-11*^∆^*^295-299 ^*(Error bars, SD. **p<0.001, *p<0.05).

## Description

SPE-11 is a sperm protein required to initiate embryonic development. Mutations of the *spe-11* gene result in a strict paternal-effect lethal phenotype. Wild-type oocytes fertilized by sperm from homozygous *spe-11* males leads to abnormal development, but the reciprocal interaction, *spe-11* mutant oocytes fertilized by wild-type male sperm, results in normal development (Hill *et al.* 1989). Sperm from homozygous *spe-11* males are morphologically normal and do not display motility defects (L’Hernault *et al.* 1988). However, the absence of functional SPE-11 results in embryonic failure at early stages. Embryonic defects include failure to complete oocyte meiosis (an event that takes place upon fertilization), formation of a weak eggshell, and improper orientation of the first mitotic spindle (Hill *et al.* 1989). In addition, Spe-11 embryos fail to complete cytokinesis during the first division resulting in multinucleated one-cell embryos that eventually die (Hill *et al.* 1989). The embryonic lethality phenotype conferred by *spe-11* mutant sperm is rescued when SPE-11 is ectopically expressed in oocytes demonstrating that SPE-11 functions during early embryogenesis and is not required during sperm development (Browning and Strome 1996). Furthermore, SPE-11 expression in oocytes did not disrupt oocyte activation because these oocytes were still capable of fertilization and did not precociously form an eggshell (i.e. prior to fertilization, Browning and Strome 1996).

SPE-11 is a 299 amino acid protein with no significant similarity to other proteins. SPE-11 is first detected in spermatocytes in the gonad of males as foci that eventually unite to form a ring around the condensed DNA of mature sperm (Browning and Strome 1996). It is predicted to be a very highly hydrophilic protein with five potential nuclear localization sequences (NLS) (Browning and Strome 1996). SPE-11 has no obvious protein domains (Browning and Strome 1996). To define functional units of the SPE-11 protein, we generated a series of GFP::SPE-11 mutant constructs along with a GFP::SPE-11 rescuing construct (Figure 1A) and created transgenic worm lines in the null *spe-11(ok2143)* background (deletion of exons two through six out of seven total exons) through microparticle bombardment. To determine functionality of each SPE-11 mutant construct we assayed the transgenic lines for SPE-11 localization in late meiotic prophase and in post-meiotic sperm and for rescue of the embryonic lethality phenotype. Similar to previous reports of SPE-11 localization, the wild-type GFP-SPE-11 construct forms foci around diplotene nuclei that eventually coalesce into a perinuclear ring in spermatids (Browning and Strome 1996) and rescues embryonic lethality (Avg. number of viable progeny=175.3, Figure 1B and 1C).

We started our mutant analysis by creating a single integrated, transgenic line carrying a mutant version of the first putative NLS (GFP::SPE-11^NLS1^). GFP::SPE-11^NLS1 ^both rescues the embryonic lethality of the *spe-11(ok2143)* mutant and localizes correctly in the male germ line (Figure 1B and 1C). We have also generated a single integrated line with the fifth putative NLS mutated (GFP::SPE-11^NLS5^). This line localizes correctly during the diplotene substage of meiotic prophase, however it fails to encircle the nuclei of post-meiotic sperm and fails to rescue the embryonic lethality of the *spe-11(ok2143)* mutant (Figure 1B and 1C). This failed rescue along with the localization defect suggests that the amino acids mutated in GFP::SPE-11^NLS5^ are important for SPE-11 function and localization. We also generated a SPE-11 construct in which three basic amino acids in the last ten amino acids of the C-terminus are changed to Ala (GFP::SPE-11^AAAmut^). Note that the GFP::SPE-11^NLS5 ^mutant has two additional basic residues changed to Ala (see Reagents section). Interestingly, the GFP::SPE-11^AAAmut^ mutant had a wild-type localization pattern, yet it completely fails to rescue the embryonic lethality of the *spe-11(ok2143)* mutant (Avg. number of viable progeny=4.6, Figure 1B and 1C).

The majority of existing *spe-11* mutant alleles truncate the SPE-11 protein suggesting that the C-terminus is very important for function. To test the importance of the C-terminus on SPE-11 function we generated an additional transgenic line, one in which only the last five amino acids are removed (GFP::SPE-11^∆295-299^). This line has a significantly lower number of viable progeny than the GFP::SPE-11 rescuing construct (p<0.05, Figure 1B). GFP::SPE-11^∆295-299 ^localization was similar to GFP::SPE-11 in diplotene stage nuclei, however, it fails to localize correctly in post-meiotic sperm (Figure 1C) suggesting that even the most C-terminal end of the protein (i.e. the last five amino acids) is essential for localization. Interestingly, while embryonic viability was severely reduced in the GFP::SPE-11^∆295-299^ line (Avg. number of viable progeny=31.9, Figure 1B and 1C), several progeny survived to adulthood.

In summary, we have identified several critical amino acids required for proper SPE-11 localization and function. The putative NLS5 and the extreme C-terminus are required for both localization and function. While not required for localization, analysis of the GFP::SPE-11^AAA^ mutant has identified three critical amino acids in the C-terminus that are required for SPE-11 function. Recreation of these alleles in the endogenous *spe-11*gene, using CRISPR/Cas9 genome editing, would be interesting to pursue.

## Methods

All strains were cultured using standard conditions (Brenner 1973). For the creation of transgenic *spe-11* animals, the promoter region, open reading frame, and 3’UTR of *spe-11* were cloned into separate entry vectors using Gateway technology (Invitrogen). These three entry clones plus an entry clone carrying GFP were recombined into a destination vector with a wild-type *unc-119* gene. Mutant *unc-119(ed3)* animals were transformed by biolistic transformation (or bombardment) with the destination clone (Wilm *et al.* 1999). Postbombardment, non-Unc animals were picked to determine which lines were integrated and which were extrachromosomal lines. At least two lines were generated for each *spe-11* construct and only integrated lines were used for analysis in this study. Lines carrying the *spe-11* transgenes were crossed into the *spe-11(ok2143)* background balanced over hT2. To visualize chromosomes, *itIs37* [mCherry::H2B] was crossed into each *spe-11(ok2143)/hT2* transgenic line. All analysis was performed on animals homozygous for *spe-11(ok2143), itIs37* [mCherry::H2B], and the *spe-11* transgene. The average number of live progeny for each strain was determined by picking single L4 hermaphrodites and transferring the animal to fresh plates every 24 h until no additional embryos were produced. The number of hatched larvae were counted after each 24-hour period. SPE-11 localization was determined through live image analysis of adult males. Adult males 24 h post L4 were anesthetized with 2 mM levamisole (Sigma), mounted on an agarose pad, and covered with a coverslip. Image collection was performed using a Nikon Eclipse E800 spinning disk confocal microscope and MetaMorph imaging software. Images were processed and analyzed using Fiji (Schindelin *et al.* 2012).

## Reagents

AG583: *spe-11(ok2143)/hT2* I; *itIs37* [*pie-1p::mCherry::his-58* + *unc-119(+)*]

AG584: *spe-11(ok2143)/hT2* I*; avIs146* [*pUNC-119(+) + spe-11p::GFP::spe-11::spe-11 3’UTR*]; *itIs37*

AG585: *spe-11(ok2143)/hT2* I*; avIs149* [SPE-11^NLS1 mutant^: P**KKK**S (amino acids 18-22) to P**AAA**S]; *itIs37*

AG586: *spe-11(ok2143)/hT2* I*; avIs152* [SPE-11^∆295-299^]; *itIs37*

AG587: *spe-11(ok2143)/hT2* I; *avIs159* [SPE-11^AAA mutant^: Sequence change: **K**FY**RK** (amino acids 289-293) to **A**FY**AA**]; *itIs37*

AG588: *spe-11(ok2143)/hT2* I*; avIs161* [SPE-11^NLS5 mutant^: Sequence change: **KK**SL**K**SVV**R**NVQ**K**FY**RK**(amino acids 277-293) to **KK**SL**A**SVV**A**NVQ**A**FY**AA**]; *itIs37*
